# Cell‐based allometry: an approach for evaluation of complexity in morphogenesis

**DOI:** 10.15302/J-QB-022-0319

**Published:** 2023-06-01

**Authors:** Ali Tarihi, Mojtaba Tarihi, Taki Tiraihi

**Affiliations:** ^1^ Department of Computer Engineering Faculty of Computer Science and Engineering Shahid Behshti University Tehran 19839‐69411 Iran; ^2^ Department of Computer Engineering Sharif University of Technology Tehran 11365‐11155 Iran; ^3^ Department of Anatomical Sciences School of Medical Sciences Tarbiat Modares University Tehran 14117‐13116 Iran

**Keywords:** embryogenesis, allometry, complexity, *C. elegans*, bioinformatics, skew matrix, morphogenesis

## Abstract

**Background:**

Morphogenesis is a complex process in a developing animal at the organ, cellular and molecular levels. In this investigation, allometry at the cellular level was evaluated.

**Methods:**

Geometric information, including the time‐lapse Cartesian coordinates of each cell’s center, was used for calculating the allometric coefficients. A zero‐centroaxial skew‐symmetrical matrix ( *CSSM*), was generated and used for constructing another square matrix (basic square matrix: *BSM*), then the determinant of *BSM* was calculated ( *d*). The logarithms of absolute *d* (Lad) of cell group at different stages of development were plotted for all of the cells in a range of development stages; the slope of the regression line was estimated then used as the allometric coefficient. Moreover, the lineage growth rate (LGR) was also calculated by plotting the Lad against the logarithm of the time. The complexity index at each stage was calculated. The method was tested on a developing *Caenorhabditis elegans* embryo.

**Results:**

We explored two out of the four first generated blastomeres in *C. elegans* embryo. The ABp and EMS lineages show that the allometric coefficient of ABp was higher than that of EMS, which was consistent with the complexity index as well as LGR.

**Conclusion:**

The conclusion of this study is that the complexity of the differentiating cells in a developing embryo can be evaluated by allometric scaling based on the data derived from the Cartesian coordinates of the cells at different stages of development.

## Abstract 2

**Author summary:** This paper addresses evaluation of morphogenesis in *Caenorhabditis elegans* based on cellular strucuture of the organism, using allometry for studying of complexity. The *x*, *y*, *z* coordinates of a cell were considered in order to generate zero‐centroaxial skew‐symmetrical matrices ( *CSSMs*) and used for constructing basic square matrices ( *BSM*s). The complexity was determined using *BSM* determinant. The results were compared with complexity index based on calculation of the branching of the mitotic tree of developing embryos, and were found to be consistent with those of *BSM*. The lineage growth rate was also evaluated.

## INTRODUCTION

*Caenorhabditis elegans* has many biological processes shared with humans and so it is a useful model for biomedical researches [1], such as in investigation of the functional characterization of a novel drug, using genomic technologies. It has also been used for investigating disease pathogenesis [2]. The favorable cost‐effectiveness of maintaining *C. elegans* assisted the investigators in using it as a model for drug action and toxicity. *C. elegans* is one of the most used biomedical models, which can be easily handled and maintained in a research laboratory [3,4].

The relationship between the weight of the brain and the weight of the body in mammals was revealed by Dubois in 1897 [5], and this was the first quantitative study of a relationship between a specific organ and the body in general. In 1924, Huxley discovered the “law of constant differential growth” [6], and Huxley and Teissier in 1936 coined the term allometry [7]. Champy reported that the relative size of the secondary sexual characteristics, as a function of body size, was related to the action of sexual hormones resulting in an increase in mitotic cell division [8,9]. This was one of the earliest attempts to correlate allometry with cellular events. Recently, Alsous *et al*. investigated morphogenesis of female germline cyst in *Drosophila* *melanogaster* by applying allometric scaling to collective cell growth and concluded that the proximity of the differentiating cells determines cell size; the investigation used cell‐based allometry [10].

Collective cell migration in the embryo was reported to be essential for successful embryo development [11]. Shellard *et al*. defined supracellular migration as a specific type of coordinated and cooperative migration, where the collective cell migration was also a cooperative and coordinated movement of groups of cells dependent on cell‐to‐cell interactions [12]. However, not all the collective migrations are supracellular ones [13]. Therefore, the relationship between collective migrations and supracellular migrations can best be explained on the basis of self‐organization, that is, the supracellular migration emerges from collective cell migration during self‐organization of a developing embryo, and the spatial order emerges spontaneously through cell‐cell interactions [14]. This suggests that both types of migration are primitive and occur during embryonic life, while during adult life the supracellular structure continues the process of differentiation, forming mature supracellular structures. A previous investigation on *C. elegans* embryogenesis at the early stages of development suggested that the random motility coefficient declined, because the EMS cells tended to regionalize sooner than those of the ABp lineage [15]. Shellard and Mayor documented that the complexity of collective migration emerged from physical and chemical communications between cells [12].

There is a consistent association between complexity and tissue growth in the morphogenesis of muscle tissue [16]. In the development of the central nervous system, prenatal and postnatal brain growth was remarkable with a high rate of neural connection, where the peak of synapse formation in monkeys from 34 weeks of gestation to 24 months after birth was 40,000 synapses per second [17]. Giagtzoglou *et al*. documented that the complexity principles were governing synaptogenesis in the nervous tissue [18], which indicated that the complexity was increasing during tissue growth. Therefore, a simultaneous quantitative evaluation of growth and complexity would be helpful in understanding morphogenesis.

Moreover, cell number and cell patterns are important parameters in evaluating complexity [19] resulting from the differential growth of various regions of the developing embryo [20]. The increase in the embryonic complexity is characterized by the increasing number of cells and their types, which contributes to the geometrical complexity of the developing organs [21]. Furthermore, a close relationship between the complexity and the scaling has been noticed, where the developing embryo was documented to follow allometric relationships [22,23]. In general, systems are classified into simple, complicated and complex according to their numbers of components, interaction and predictability [24]. Coveney reported that a complex system was essentially composed of a collection of simple units interacting with each other, emerging into an unpredictable system [25]. Prediction could be achieved, however, by modeling the system [26]. West and Brown reported that a complex system in biology had hierarchical organization [27], which could be scaled from the molecular level to higher orders such as the organ and the whole body. Allometric scaling, is a scaling method describing the mathematical relationship between numerous structural or physiological parameters as a function of body weight [28]. It is one of the methods that can be used in evaluating scaling in biology. For example, gene networks and gene expression have been considered as important aspects in pattern formation of a developing embryo [29], where complex molecular interactions can contribute to a complex pattern formation [30]. Wolpert and Lewis also reported that the genetic network accounted for positional information and pattern formation in a developing organism [31].

Our method is based on estimating the allometric parameters from data derived from the three dimensional Cartesian coordinates of the cells forming the developing embryo using captured time‐lapse images, in order to examine the possible use of the allometric coefficient as a measure for evaluating complexity. Also, the work investigates whether there is any relationship between cell growth and complexity by quantifying them by the lineage growth rate and the complexity index, respectively.

## RESULTS

### Data source

**Figure 1:**
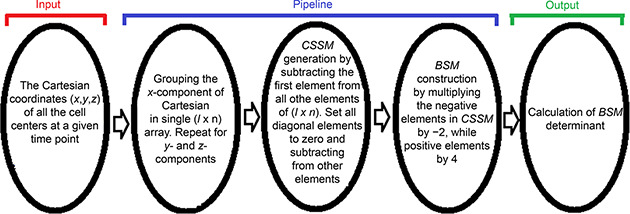
**Pipeline of data handling.** It shows different steps of data input (The components of the Cartesian coordinates of *C. elegans* embryo cells), data processing and calculation output used in generating the zero‐centroaxial skew‐symmetrical matrix (*CSSM*) and constructing the basic square matrix (*BSM*).

**Figure 2:**
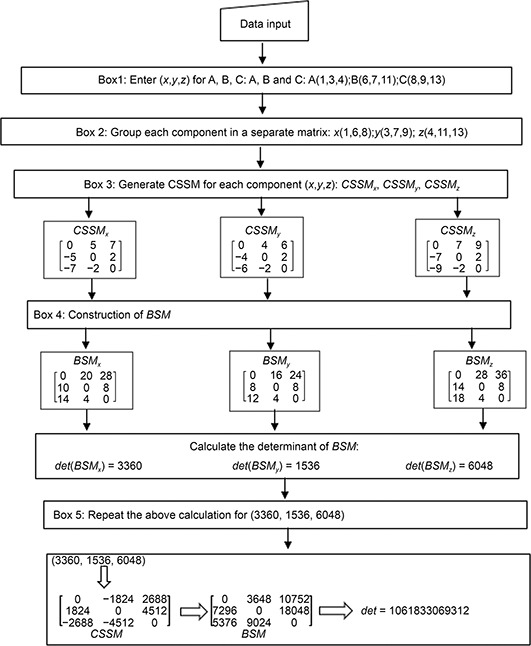
**A detailed flow chart for matrix calculation.** It shows the flow chart of the zero‐centroaxial skew‐symmetrical matrix (*CSSM*) generation, the construction of the basic square matrix (*BSM*) and the determinants calculation. Box 1: Enter (*x,y,z*) of the cell centers for all cells in a given A, B and C developmental stage. Example: embryo at 3‐cell stage; A, B and C: A(1,3,4); B(6,7,11); C(8,9,13) Box 2: Group the *x*‐components in (1×*n*)*_x_* matrix. Also, group the *y*‐ and *z*‐components: (1×*n)_y_* and (1×*n)_z_* matrices, respectively. The (1×*n*) matrices were (1,6,8), (3,7,9) and (4,11,13), respectively. Box 3: Generate *CSSM* for each component (*x,y,z*): *CSSM_x_*, *CSSM_y_*, *CSSM_z_* by subtracting the first element in the first row from the elements in the row resulting in generation of the first *CSSM* row. In the second row, we subtract the second element from all of the elements in the first *CSSM* row, the diagonal element is zero; this was the second row of *CSSM*. In the third row, we subtract the third element from all of the elements in the second row; the diagonal elements were zero, and this was the third row of *CSSM*. For higher order of *CSSM*, this procedure can be repeated. Box 4: Construct *BSM_x_*, *BSM_y_* and *BSM_z_* by multiplying *CSSMs* negative element by ‐2 and the positive element by 4. Box 5: Enter the values of *det(BSM_x_)*, *det(BSM_y_)* and *det(BSM_z_)* as (1×*n*) matrix, generate *CSSM*, constructe *BSM* and calculate the final determinant as above.

Fig.1 shows the pipeline of data input, processing and output; the details are explained in the materials and methods section. The captured images with their level of focusing were used to estimate the center of the cells where the components of the Cartesian coordinates ( *x,y,z*) of each cell were determined; the related development times of *C. elegans* are explained in the materials and methods section. The Cartesian coordinates of each cell center were used in the study as indicated in the pipeline of the data input, processing and calculation of the output (Fig.1). The details of the analysis are explained in Fig.2.

### Simple and complex pattern

**Figure 3:**
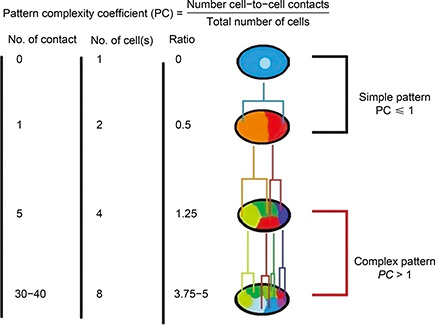
**A simple pattern compared with a complex one.** It shows sketches of the simple and complex patterns in early stages of the developing embryo in *C. elegans*.

Fig.3 shows an example of a complex pattern based on the estimation of the pattern complexity coefficient. This is a simple estimate of the cell arrangement at the early stages of embryo development.

### Generation of zero‐centroaxial skew‐symmetrical matrix ( *CSSM*) and construction of *BSM*

The generated *CSSM* can essentially be area (2×2) , volume (3×3) or hypervolume (n×n) , where *n* is more than 3. The *n*‐dimensional matrix can be geometrically considered as parallelepiped and be represented by a (n×n) matrix. The volume of the parallelepiped is equal to the determinant of a (3×3) matrix, while the determinant of n×n represents the size of an *n*‐dimensional hyperparallelepiped [32]. One of the parameters that can be used in evaluating allometry is the volume [33], where we can use a (3×3) matrix as a geometrical value in calculating the allometric coefficient. The volume can be extended to hypervolume (n×n) in evaluating the allometric scaling [34,35].

**Figure 4:**
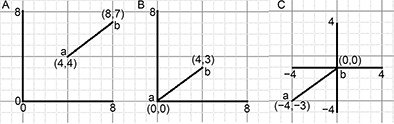
**An example of the zero‐centroaxial skew‐symmetrical matrix generation.** It represents a simplified model for generating the zero‐centroaxial skew‐symmetrical matrix (*CSSM*). (A) presents a straight line (a,b: can be assumed to be the centers of two neighboring cells). The two‐dimensional Cartesian coordinates are: a(4,4) and b(8,7), respectively. (B) represents the translation of point a to the origin of the Cartesian coordinates (OCC), where the new dimension of a and b are (0,0) and (4,3), respectively. (C) represents the translation of point b to the OCC, where the new dimension of a and b are (−4,−3) and (0,0), respectively. The generation of the *CSSM* and construction of the basic square matrix (*BSM*) are presented in the materials and methods section.

Fig.4 shows an example of *CSSM* generation in a 2‐dimensional space (2×2) matrix. In this study, we use the allometric coefficient for estimating the complexity by generating a (n×n) *CSSM* matrix, where *n* represents the number of cells entered in the calculation of the allometric coefficient. Tab.1 shows a line with two points (a, b) at its ends, the Cartesian coordinates of the first and the second points are (4,4) and (8,7), respectively. We subtract 4 from 8, and 4 from 4 (this operation is a geometric translation of ab line to the origin of the Cartesian coordinates). Therefore, the new coordinates for a and b will be (0,0) and (4,3). The row of the *x‐* and *y‐*coordinates are (0,4) and (0,3), respectively.

The generated *CSSM* and the constructed *BSM* for the *x‐* and *y‐*coordinates are represented in Tab.1 and Tab.2.

**Table 1:** The zero‐centroaxial skew symmetric matrix (*CSSM*) generation and the construction of the basic square matrix (*BSM*) at the *x*‐component

	*CSSM*_*x*_		*BSM*_*x*_	
Translation to OCC	a	b	a	b
First translation	0	4	0	16
Second translation	−4	0	8	0

*CSSM* is a zero‐centroaxial skew‐symmetrical matrix, *BSM* is a basic square matrix and OCC is the origin of the Cartisean coordinates. Tab.1 shows The *CSSM* and the *BSM* related to the *x*‐components (*CSSM*_*x*_) of a and b points in the straight line shown in Fig.4. The first row represents the values of the *x*‐components of a and *b* points on the line following the translation of point a (first translation) in Fig.4 to the origin of the Cartesian coordinates (OCC) (Fig.4), The values in the second row is generated from the points a and b on the same line following the translation of point b (second translation) in Fig.4 to OCC (Fig.4). The *BSM*_*x*_ (the *BSM* related to the *x*‐components of the straight line in Fig.4) was constructed by multiplying the negative element of *CSSM*_*x*_ by −2 and the positive element by +4.

### Complexity index

**Figure 5:**
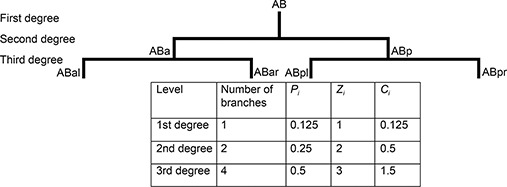
**An example of the complexity index calculation.** It shows an example of the complexity index calculation using the morphogenesis of AB blastomere in *C. elegans* (the first level or degree of complexity); ABa and ABp (the second level or degree of complexity), where each daughter cell formed a branch. ABa is divided into ABal and ABar, and ABp which is divided into ABpl and ABpr (the third level or degree of complexity). The table in this figure shows the number of branches at each level (*p_i_*: branching coefficient, *z_i_*: branching level coefficient *C_i_*: complexity coefficient). The summation of *C_i_* is the complexity index (2.125).

Fig.5 shows an example of the complexity index calculation using the morphogenesis of AB blastomere in *C. elegans* (the first level or degree of complexity); ABa and ABp (the second level or degree of complexity), where each daughter cell formed a branch. ABa divided into ABal and ABar, and ABp which is divided into ABpl and ABpr (the third level or degree of complexity). The table in this Fig.5 shows the number of branches of *C. elegans* morphogenesis tree at each level or degree ( *p*_*i*_: branching coefficient, *z*_*i*_: branching level coefficient *C*_*i*_: complexity coefficient). The summation of *C*_*i*_ is the complexity index (2.125).

**Table 2:** The zero‐centroaxial skew‐symmetrical matrix (*CSSM*) generation and the construction of the basic square matrix (*BSM*) at the *y*‐component

	*CSSM*_*y*_		*BSM*_*y*_	
Translation to OCC	a	b	a	b
First translation	0	3	0	12
Second translation	−3	0	6	0

*CSSM* is a zero‐centroaxial skew‐symmetrical matrix, *BSM* is a basic square matrix and OCC is the origin of the Cartisean coordinates. Tab.2 shows the *CSSM* and the *BSM* related to the *y*‐components (*CSSM*_*y*_) of a and b points on the straight line shown in Fig.4. The first row represents the values of the *y*‐components of a and b points in the line following the translation of point a (first translation) in Fig.4 to the origin of the Cartesian coordinates (OCC) (Fig.4). The values in the second row are generated from the points a and b on the same line following the translation of point b (second translation) in Fig.4 to OCC (Fig.4). The *BSM*_*y*_ (the *BSM* related to the *y*‐components of the straight line in Fig.4) was constructed by multiplying the negative element of *CSSM*_*x*_ by −2 and the positive element by +4.

Tab.3 and Tab.4 show the parameters of calculating the complexity index of the ABp sublineage, which are higher than those of the EMS. They are 10.031 and 8.417.

### Allometric analysis

**Figure 6:**
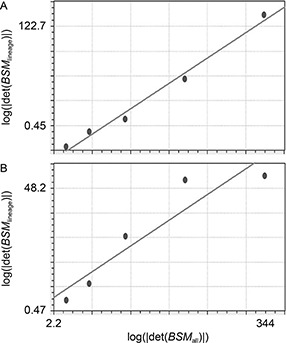
**Results of the allometric coefficients calculations.** It represents the calculations of the allometric coefficients of the ABp and the EMS descendent cells using linear regressions, where the logarithms of the absolute values of the basic square matrix determinants at 30, 55, 82, 109, and 123 min stages (abscissa) of all of the cells at each stage are plotted against the logarithms of the absolute values of the basic square matrix determinants of the lineage (ordinate) at different time points of the ABp descendent cells (A) and the EMS descendent cells (B) at each stage. The regression line of the ABp descendent cells is *y* = −9.3 + 0.44*x* (standard error = 7.15 and correlation coefficient = 0.99), while in the EMS descendent cells *y* = 5.42 + 0.18*x* (standard error = 9.19 and the correlation coefficient = 0.93).

**Figure 7:**
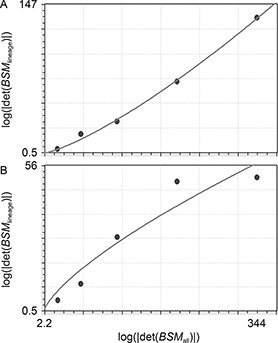
**Nonlinear curve fitting for the ABp and EMS descendent cells.** It represents calculations of the nonlinear curve fitting for the ABp and EMS descendent cells using nonlinear regressions (the power fit model). The logarithms of the absolute values of the basic square matrix determinants at 30, 55, 82, 109, and 123 min stages (abscissa) of all the cells at each stage are plotted against the logarithms of absolute values of the basic square matrix determinants (ordinate) at different time points of the ABp descendent cells (A) and the EMS descendent cells (B). The power fit model in the ABp descendent cells is *y* = 0.073*x* ^1.31^ (standard error = 3.27 and correlation coefficient = 0.999), while in the EMS descendent cells *y* = 0.94*x* ^0.72^ (standard error = 7.68 and the correlation coefficient = 0. 954).

Fig.6 demonstrates the plotted data of the logarithm of the absolute values of the *BSM* determinant (log⁡|det(BSM)|) of the cells in a lineage versus all cells in the embryo at a given stage, where the linear regression was estimated, the *b* coefficients (allometric coefficient) of the ABp and the EMS are 0.44 and 0.18, respectively. A nonlinear curve fitting was also done using the power fit model, and the data show that there is a consistency with those of allometric coefficients ( Fig.7).

**Table 3:** Complexity index calculation of ABp sublineage

Level of complexity	Number of branches	*p*_*i*_ coefficient	*z*_*i*_ coefficient	*C*_*i*_ coefficient
1st degree	1	0.0313	1	0.0313
2nd degree	2	0.063	2	0.125
3rd degree	4	0.125	3	0.375
4th degree	8	0.25	4	1
5th degree	16	0.5	5	2.5
6th degree	32	1	6	6

*p*_*i*_: number of branches at each level (branching coefficient), *z*_*i*_: branching level coefficient and *C*_*i*_: complexity coefficient. The summation of *C*_*i*_ is the complexity index (10.031).

**Table 4:** Complexity index calculation of EMS sublineage

Level of complexity	Number of branches	*p*_*i*_ coefficient	*z*_*i*_ coefficient	*C*_*i*_ coefficient
1st degree	1	0.0833	1	0.083
2nd degree	2	0.1667	2	0.333
3rd degree	4	0.333	3	1
4th degree	6	0.5	4	2
5th degree	12	0.1	5	5

*p*_*i*_: number of branches at each level (branching coefficient), *z*_*i*_: branching level coefficient and *C*_*i*_: complexity coefficient. The summation of *C*_*i*_ is the complexity index (8.417).

### Lineage growth rate

**Figure 8:**
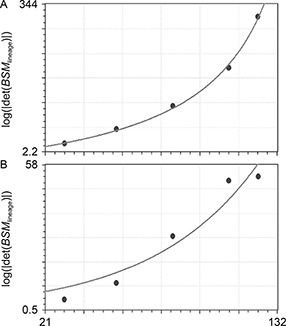
**Results of calculations of the lineage growth rate.** It represents the calculations of the lineage growth rate of the ABp and the EMS descendent cells using an exponential model. The logarithms of the absolute values of the basic square matrix determinants at 30, 55, 82, 109, and 123 mins stages (ordinate) of all of the cells in each lineage at each stage are plotted against time (in minutes: abscissa) at different time points of the ABp descendent cells (A) and the EMS descendent cells (B). The exponential model of the ABp descendent cells is *y* = 1.47e^0.036*x*^ (standard error = 6.692; correlation coefficient = 0.994), while that of the EMS descendent cells is *y* = 5.2e^0.02*x*^ (standard error = 6.66; correlation coefficient = 0.966).

The logarithms of the absolute values of the *BSM* determinants in the ABp and the EMS were plotted against the time points (30, 55, 82, 109 and 123 mins), and the exponential model was fitted, the rate of growth was higher in the ABp than in the EMS (0.036 and 0.02, respectively; Fig.8 and B).

## DISCUSSION

A complex system is composed of several heterogeneous parts. When all these parts are isolated, they cannot give the idea of the complexity; when these parts are combined together, they may represent the complex system. Therefore, simple summation of these parts fails to represent the whole complex system, they should interact in a proper order and a precise hierarchical assembly [36]. In other words, the complex system is not a simple linear assembly of the parts. A complex system, particularly in biology, can be represented as a non‐linear mathematical combination of all the parts [37]. In previous research, we reported the physical displacement of the cells resulting in regionalization of EMS and ABp sublineages [15]. Schnabel *et al*. explained that a new mechanism for morphogenesis formed a new pattern as the cells moved to a new position [38]. Cell movement during morphogenesis is orchestrated in time and space in order to reshape the embryo [39], while cell‐generated mechanical forces play a critical role in generating complex morphogenesis.

### Matrix generation and analysis

If a *CSSM* has (n×n) dimensions with *n* as an odd number, the property of this matrix will be a singular matrix with a determinant value equal to zero [36]. In order to obtain non‐zero values of the determinants for all the generated matrices (odd and even), the transformation operation (multiplication of *CSSM* positive elements with 4 and negative elements with −2) was applied resulting in constructing the basic square matrix ( *BSM*) [4], which satisfies the properties of a square matrix [37,38]. It must be emphasized that a (1×n) matrix should not have 2 or more elements with identical values.

The *BSM* was constructed as explained in the materials and methods section. The determinant of the latter was used for further calculation of the allometric coefficient.

At the early stage of development, there are fewer cells. Thus, the *BSM* had smaller value. As the embryo develops, the number of cells increases, and so do the number of the elements in the matrix, the size of the *BSM* and the absolute value of the logarithm of absolute value of *BSM* determinant [38]. The latter value was used in evaluating the trend of the increase in the number of cells as the developing embryo grew [39].

The advantage of using the *CSSM* and *BSM* method is the possibility of applying it to a system with actively increasing dimensions, where the number of cells at the next developmental stage tends to be higher than the previous one. The model is flexible in adapting higher points which increase exponentially during development as in the case of multidimensional data.

This method is a general one that can be used for different morphogenetic and geometric parameters, and other physical variables. We applied the cell center (center of gravity or centroid) in order to investigate self‐organization, complexity and lineage growth rate. Other data such as mathematical functions can be used instead of the cell center, such as single cell motion, cell diffusion, and other parameters including those reported by Cao *et al*. [40]. At the molecular level, the data of gene expression [40] and signaling [41] can be studied using the *CSSM* method. Each cell can be represented by a defined mathematical function. For instance, if we have 3 cells, then we have *f*( *x*_1_), *f*( *x*_2_), *f*( *x*_3_), where *f*( *x*) is the function of a cell at a given developmental stage. The values of a given parameter of these cells can be used for generating *CSSM* and constructing *BSM*.

In this investigation, we tried *CSSM* technique as an example for quantitative evaluation of metazoan development, for example, it can be employed in other datasets such as those reported by Guan *et al*. who used more than 200 *C. elegans* embryo samples with time‐lapse cell positions recorded up to 350‐cell stage, which can make further analysis much more systematic, statistical, and reliable [42,103,123] compared with only one embryo as investigated in this paper. Moreover, this technique can be applied for other species in order to analyze their cell lineages such as the cell position data in ascidian embryogenesis [43].

### The complexity index

The use of the complexity index for assessing embryo development is an approach to evaluating the trend of development history of an embryo, where the change of the complexity index in an organism is a function of its developmental stage [23].

The complexity index has been used in characterizing the trend of changes in genome complexity, dendritic complexity in the neurons, and the wave of electrocardiogram, and has been applied in epilepsy study, pharmacology and other fields [44, 45, 46, 47, 48]. For example, the branching order was commonly used in neuroanatomy so as to evaluate the complexity of the neurons with dendrite branching; the method requires information about the terminal tips and the dendrite lengths in order to estimate the complexity index [49,50]. However, in this investigation, the whole body complexity is not estimated because the main focus is on evaluating and comparing the complexity of a sublineage within the whole body of the developing embryo with that of another sublineage. Therefore, the technique used in this investigation tracks the point of cell division (bifurcation points) in each lineage, while the method used by Richter and Splechtna can be applied for determining the complexity index of a sublineage within the whole body of the developing embryo [51]. The results for the complexity index in this investigation are consistent with that of other investigators [52,53]. Therefore, the results show that a higher value of the complexity index means the lineage structure is more complex *.* Burggren documented that the intermediate developmental stage had higher complexity than the later stages of development [23].

Moreover, by definition, lineage complexity is characterized by increase in the number of somatic cells], generated by either asynchronous or synchronous division, with an increase in diversity [54. The complexity of those generated by asynchronous division is greater than that of the ones generated by synchronous division [55].

### Allometric analysis

One of the well‐known measures of complexity is the fractal dimension [24,56, 57, 58, 59]. In a previous research [4], we reported that the scale‐invariant power law ( *SIPL*) coefficient was consistent with the fractal dimension. The *SIPL* coefficient of the ABp sublineage was higher than EMS sublineage in the developing *C. elegans* embryo. However, the values were very close (ABp = 1.342 and EMS = 1.339); therefore, it is useful to find another measure for estimating the complexity (instead of the *SIPL* coefficient) using the Cartesian coordinates (3‐dimensional) derived from the positional information (the same data set used in calculating the *SIPL* coefficient) and allometry. Non‐linear allometry shows better results in separating these sublineages on the basis of complexity. In this study, the coefficients estimated by nonlinear curve fitting showed better results than that of the linear regression (the values for ABp and EMS were 1.31 and 0.72, respectively).

Yamamoto *et al*. reported that the lower the fractal dimension the less complex the system was; we can conclude that the lower allometry value in the EMS means that it is less complex compared with ABp [60]. Moreover, the measure used in this study is intended to calculate the complexity based on the allometric coefficient, which is more systematic and is used extensively in biology [61, 62, 63, 64, 65, 66, 67, 68, 69, 70, 71, 72, 73, 74, 75, 76, 77, 78, 79, 80, 81, 82, 83, 84, 85, 86, 87, 88]. Burggren revealed that physiological complexity increased with embryonic development [23], which followed the allometric law; allometric growth was confirmed in the development of zebrafish [89]. Other investigators reported differential growth with different allometric coefficients among different parts of the human brain [24,90,91].

The complexity index of the ABp was higher than that of the EMS, it was reported that the complexity increase was higher in the larger organs than in the smaller ones [92]. Rosas and Bastir’s finding supported the hypothesis that allometry contributes to the organization of variation in complex morphological structure [93]. In fact, the data of both the ABp and the EMS derived cells show nonlinearity, where the curve fitting showed a higher coefficient in the ABp derived cells compared with that of the EMS [94,95]. Moreover, the complexity had a genomic level, where the gene network determined the morphology [96]. Therefore, quantifying the complexity using cell based‐allometric analysis during morphogenetic process may be a useful means for finding correlation between cellular and molecular levels [97].

Johnston *et al*. documented that the conventional method for calculating the allometric coefficient was based on the relative increase in weight or volume of an organ to the whole body [16]. In this study, the calculation is based on the relative changes in cell arrangement and number, where the cell is considered as the basic unit of tissues and organs. The changes in the developmental stages essentially rely on the changes in the cellular components forming the developing organ [20]. In a developing embryo, the increase in cellularity is small, characterized by relatively small increases in the mass, therefore, it is difficult to obtain accurate weight measurements for very small organs in the embryo [98].

During the early stages of embryogenesis in metazoa, the cells are arranged into a specific pattern forming the body tissues [99]. Cellular rearrangement during embryonic development results in establishing three germ layers in the embryo [100]. The changes in the patterns result from gradual allometric growth, which is linked to gene expression, the generation of morphological variation is based on allometric growth change [101]. On the other hand, Zhao *et al*. documented that *Caenorhabditis briggsae*, closely related species to *C. elegans*, showed that the change in cell division time could change the cell position, this indicates that the positional information depends on temporal events [102]. Other investigators compared both the nematodes division‐timing program and the cell‐arrangement pattern; they found that there were variations in the positional information within a single species as well as between the two species [103]. The position of the cells in each lineage during internalization correlates well with the antero‐posterior position suggesting that gastrulation plays a large part in positioning the cells [104]. Guan *et al*. reported several mechanical models proposed for the development of the embryo prior to gastrulation confirming that cell division timing was accurately regulated. Whereas in gastrulation, cell internalization was documented by Pohl *et al*. [105], it was associated with an increase in cell number and decrease in cell size; in *C. elegans*, the cells contacted the eggshell and became closer to each other, whereas the E2 cell was the largest cell inside the embryo [106]. The E lineage has an extended cell cycle compared to that of the MS lineage [107]. We used differential lineage growth rate to evaluate the migration of the cells during the development in order to study cell displacement during gastrulation. Cell internalization during gastrulation due to mechanical forces is associated with morphogenesis [108].

Marcus *et al*. revealed that there were three different levels of allometry: static, ontogenetic and evolutionary. The ontogenetic one was due to the growth process, and they mentioned three types of data used in studying it: longitudinal, cross‐sectional and mixed types [109].

Moreover, theoretical studies showed that various models of growth involved functions of time with simple allometric properties [109]. Brown documented that an embryo or part of an embryo could be scaled up allometrically so as to predict its functionality [110]. Katz used allometry in order to describe relative growth using the relative number of cell divisions [111]. Also, in mammalian brain allometry, the high prenatal allometric coefficient of the individual ontogenetic trajectory declined at post‐natal life; this change in the ontogenetic curve corresponds to the cessation of neuronal division [98]. In this study, we used ABp and EMS sublineages in order to demonstrate their differential growth, which can be interpreted as being due to competition among growing organs resulting in changes in their morphological asymmetry [112]. This intra‐organism plasticity is important in giving rise to intraspecific allometry [113], while the ontogenetic variation caused by the differential growth of the intra‐organism organs leads to interspecific allometric changes [98,114]. Most of the phylogenetic changes that take place can be explained by the change in the developmental timing during the evolution [115].

Steiner *et al*. reported that allometric scaling showed a shift in the growth rate of a given structure in the embryo [116]. The Cartesian coordinates of a given cell are the positional information, which can be used as a function of another variable, such as the change in gene expression during the development process. In fact, Stern and Emlen reported that allometry was a function of cells forming a contiguous field expressing the same genes [78]. Therefore, the allometric function can incorporate the positional data with genetic information. However, the relationship between the genetic information and allometry is poorly understood, because of the lack of the tools required for the analysis [97]. However, a *CSSM* can be a useful tool in the analysis of the developing embryo.

The cell division of endoderm progenitor cells in *C. elegans*, Ea and Ep, is asynchronous with a significant delay of cell division time. A molecular study showed that molecular events were required so as to coordinate the differentiation, cell division timing and cell migration in order to ensure proper development [117]. On the other hand, cell size is considered as a critical factor for cell cycle regulation, in *C. elegans*, the relationship between cell cycle duration and cell size exhibited a power law, where the cells can be grouped into 3 classes (based on cell radius): 1‐highly size‐correlated, 2‐moderately size‐correlated and 3‐potentially size‐non‐correlated. Moreover, the ratio between the nucleus volume and cell volume exhibited a power law relationship in those with size‐correlated classes, and showed that the relationship between the cell cycle length and cell volume was allometric in nature [118].

In fact, size scaling in developing and growing *C. elegans* embryo was reported as hierarchical in nature; for example, the relative size of nucleolus is predictive of the growth rate of the entire worm, and the growth of individual structures is differentially regulated during development [3,119]. Hara and Kimura reported that the mitotic spindle width of various cell sizes and spindle length during embryogenesis had allometric relationship with ploidy in *C. elegans* embryos [119]. Moreover, an allometric equation has been given that has described spindle width as a function of the length of the hypotenuse of the spindle as well as nuclear ploidy [119]. Also, Needleman confirmed that there was a correlation between cell size and the behavior of the cytoskeletal division machinery during embryogenesis [120]. At the molecular level, metalloprotease (mig‐17) is part of the muscle‐epidermis‐glia signaling axis that sustains synaptic specificity during the organism’s allometric growth [121].

On the other hand, the mechanical properties of a tissue are critical for embryonic development and tissue form [122]. Embryonic development is a precise and complex process involving mechanical forces interacting in space and time [123]. Besides, Solnica‐Krezel and Sepich documented that the cytoskeleton was an essential component in gastrulation [124]. The mechanical stiffness of the local tissue environment and the contractile activity of the cells contribute to morphogenesis. Stiffness and contractility are both involved in the cellular mechanical stresses that are essential for mechanotransduction [125].

### Lineage growth rate

In this study, the uniexponential model is used in evaluating the growth rate [126], the results show that the growth rate was higher in the ABp sublineage than the EMS one (0.036 and 0.02, respectively) ( Fig.8). The growth rate in *LGR* was consistent with our previous work, which was based on the diffusion coefficient (the diffusing particles are the cells) [53].

The plot of the logarithm of the absolute values of the *BSM* determinant as a function of time shows that the growth of the ABp and the EMS descendant cells was exponential in nature, which is consistent with Goedbloed’s findings of the exponential growth of rat and mouse embryos [127]. In addition, Luecke *et al*. reported the embryo body growth at the early stage was exponential [128]. The results also show a higher growth rate in the ABp derived cells than those of the EMS ones, where the number of cells in ABp exceeded that of EMS. The analysis of the first 4 stages show that the rate was higher in the EMS derived cells than those of the ABp ones and that the trend was reversed when all the stages were used in the study. This is consistent with our previous study, where there was a rapid diffusion at the early phase in the EMS lineage followed by a slower one [53].

Sbarbati and Strackee reported that during organogenesis, there was an initial exponential model, where differential growth was noticed in the growth of different tissues, where the growth of stomach epithelium was faster than that of the mesenchyme [129]. Cowan and Morris reported that the growing embryo had initially an exponential growth phase then the differentiating cells departed from this growth mode [130].

Growth, patterning and morphogenetic movements are components of the development process in neural plate, resulting in three‐dimensional complexity of the CNS [131]. Growth, quantified by *LGR*, is a factor in the developing embryo that contributes to complexity. The results show that the complexity index and the allometric coefficient are higher in the ABp sublineage than in the EMS one. Furthermore, the evaluation of patterning in the development requires special attention because a field of mathematics, the science of patterns, has been emerging, where the mathematician identify and analyze abstract patterns such as numerical patterns, patterns of shape, patterns of motion, patterns of behavior, voting patterns in a population, patterns of repeating chance events, and others [132]. While abstracting the morphogenesis can be helpful in understanding the development process, pattern formation is also an important feature of morphogenesis [133]. On the other hand, the generation of a complex pattern contributes to morphogenetic movements with involvement of signaling and responding genes [134]. In fact, morphogenetic movements during embryo development were reported to be controlled by molecular mechanisms such as morphogens including Wnt/β‐catenin, sonic hedgehog, and fibroblast growth factor. Also, transcription regulators such as myogenic regulatory factors (MRFs: myoD, myf5, mrf4/herculin/myf6, and myogenin), myogenic differentiation (myoD), myogenic factor 5 (myf5), myogenic regulatory factor 4 (mrf4) and myogenin (myog) are involved in muscle development [135]. Both cellular and molecular mechanisms coordinate the morphogenesis process [136]. Therefore, abstraction of the huge molecular and cellular morphogenesis data can be useful in understanding the development of the embryo, thus using *CSSM* particularly in the current state of knowledge where the morphogenesis database is growing rapidly [137, 138, 139, 140].

### Simple versus complex patterns

Pattern formation in the embryo can occur either by cellular movement [141] or differential growth [142]. Savageau revealed that morphogenesis was a complex process originating from differential growth and was essentially allometric in nature [20,143]. On the other hand, cellular movement was studied by many techniques, such as vector analysis [144], random motility coefficient and the diffusion coefficient [53,145]. In this study, the cellular movement is evaluated by a *CSSM* in three dimensions, while most of the previous investigations were done in two dimensions [144]. In addition, Tian *et al*. reported that combining mechanical forces contributed to the order and orientation of cell division and ensured robust arrangement of the cells as well as pattern formation. This simplified mechanical model can simulate the arrangement of cells, such as in investigation of self‐organization in early nematode embryogenesis [146]. In this study, we also evaluate the pattern complexity coefficient in order to demonstrate the difference between simple and complex patterns. The pattern complexity coefficient is based on the ratio between the number of cell‐to‐cell contact and the total number of cells at a given stage of embryonic development. This is a simple approach for evaluating cell patterning. However, the formula can be refined for evaluating quantitative systematic patterning. Siegenfeld and Bar‐Yam revealed that two factors contributed to the generation of a complex system: branching and interaction among its parts [147]. Fig.3 represents early stages of *C. elegans* morphogenesis, where the embryonic cells divide dichotomously with an increase in the morphogenesis tree of the embryo, which resulted in an increase in the number of dividing cells and their interactions [148]. There is a proportional increase in the number of cell‐to‐cell contacts during morphogenesis, where the zygote has zero contact. This represents the simplest pattern. When the zygote divides into AB and P1, there is a single contact. After division of AB and P1 into 4 cells, there were 5 contacts. At the 8‐cell stage, the number of contacts is 30−40. The 2‐cell stage had 1 contact, and the 3‐cell stage had 3, which represents a simple pattern [149]. The ratio of the cell‐to‐cell contact to the cell number was calculated; if the ratio was ≤1, then the pattern was simple, otherwise the pattern was complex. The more branching there is in the morphogenesis tree, the more interaction (cell‐to‐cell contact) there is, and the more complex is the morphogenesis pattern.

### A possible relationship between cell movement and complexity

In a previous study, we analyzed the cell movement in *C. elegans* embryo and the quantitative evaluation confirmed that the *in vivo* cellular movement was non‐random and this movement resulted in regionalization of the cells. In the next step, we used a scale‐invariant power law study and the results indicated that self‐organization was the main mechanism in the morphogenesis of a developing embryo and that complexity is the main feature of the developing embryo ( Tab.5).

## MATERIALS AND METHODS

The geometric information, including the time‐lapse Cartesian coordinates of each cell’s center was used for calculating the allometric coefficients. We explored two out of the four first generated blastomeres in *C. elegans* embryo, the ABp and EMS lineages, showing that the allometric coefficient of ABp was higher than that of EMS ( Fig.6). Moreover, the selection of ABp and EMS lineages out of the four founder cells was based on the fact that ABa, ABp, and EMS were somatic founder cells that kept dividing and generating new cell fates while ABa and ABp were highly similar. So comparing ABp and EMS was sufficient to demonstrate the usability of the proposed methods.

**Table 5:** A possible relationship between cell movement and complexity, and the purpose of each event (cell motion, regionalization and self‐organization) that occurred during morphogenesis in *C. elegans* embryo, and the mechanism of that event as well as the tool used in the analysis

Event	Tool	Outcome	Refs.
Cell movement and migration	Statistical approach for (random motility method and diffusion)	Non‐random (regionalization)	[15]
Regionalization	Scale‐invariant power law approach	Self‐organization	[4]
Self‐organization	Allometric approach	Complexity	Current

### General methodology

#### Generation of a zero‐centroaxial *CSSM*

The data acquisition was performed by using the time‐lapse Cartesian coordinates (x,y,z) of each cell’s center at a given time point (30, 55, 82, 109 and 123 mins) of *C. elegans* embryo. These time points were applied for two out of the four first generated blastomeres in *C. elegans* embryo, the ABp and EMS lineages.

The captured images with their level of focusing and the related development times of *C. elegans* embryo were documented by Schnabel *et al*. [141]. The level of focusing for each cell (captured time‐lapse images) was recorded. Several boundary points on the nuclear boundary of a chosen cell profile and the centroid of the largest nuclear profile was estimated (the *x* and *y* components of the centroid). The geometrical features (centroid) were estimated using imageJ software in NIH website. The level of each cell and the largest profile in a given cell was considered as a *z*‐axis of the Cartesian coordinates. The Cartesian coordinates of the cell centers are indicated in the pipeline of data input, data processing and calculation output, see Fig.1, and details of the analysis are explained in Fig.2.

The Cartesian coordinates of the three‐dimensional Euclidean space [150,151] ( *x*, *y*, *z*) are used for generating *CSSM*. The *x*‐components of the Cartesian coordinates of the cells are $\left(x_{i}\right)$ , where *i* = *n*; ($x_{1},x_{2},x_{3},\ldots ,x_{n}$ is related to *cell*_1_, *cell*_2_, *cell*_3_,…, *cell*_*n*_). The 1×n array for the *x*‐component is $\left(x_{1},x_{2},x_{3},\ldots ,x_{n}\right)$ . It should be emphasized that there should not be two or more elements with identical values, and if so then only one of them should remain. Similar matrices for *y* and *z* components are generated, $\left(y_{1},y_{2},y_{3},\ldots ,y_{n}\right)$ and $\left({\text{z}}_{1},{\text{z}}_{2},{\text{z}}_{3},\ldots \right)$ , respectively. The (1×n) matrix is entered in order to generate *CSSM*. The latter is used in constructing the basic square matrix ( *BSM*), then its determinant is calculated. Accordingly, the calculation is also done for the *y*‐ and *z*‐components. The determinants of *x*‐, *y*‐ and *z*‐components are used for estimating the allometric coefficient and the lineage growth rate. Fig.4 demonstrates the Euclidean space of the Cartesian coordinates of ab line. Fig.4 shows the *x*‐ and *y*‐components of a and b points before they were translated (assuming they were the centers of two neighboring cells). Fig.4 represents the *x*‐ and *y*‐components of a and b points after translating point a to the point of the origin of the Cartesian coordinates ( *OCC*). Subsequently, point b was translated to point OCC ( Fig.4). There were two *CSSM* related to the *x*‐ and *y*‐components of the Cartesian coordinates of points a and b. The *CSSM* was generated by arranging the *x*‐components of a and b as the first row of *x*‐ *CSSM* in Fig.4. The second row was related to the *x*‐components of Fig.4 following the translation of point b to OCC. The generated matrix was a skew square matrix with zero diagonal ( Tab.1). The *x*‐ *CSSM* was ${CSSM}_{x}=\left[\begin{array}{cc} 0 & 4\\ -4 & 0 \end{array}\right],$ while the *y‐CSSM* was ${CSSM}_{y}=$ $\left[\begin{array}{cc} 0 & 3\\ -3 & 0 \end{array}\right]$ .

#### Construction of the basic square matrix ( *BSM*)

The *BSM* was constructed by multiplying the negative element of *CSSM* by −2 and the positive one by +4. The *x‐BSM* was ${BSM}_{x}=\left[\begin{array}{cc} 0 & 16\\ 8 & 0 \end{array}\right],$ while the *y‐BSM* was ${BSM}_{y}=\left[\begin{array}{cc} 0 & 12\\ 6 & 0 \end{array}\right],$ and the determinants were −128 and −72, respectively. In fact, a second algorithm could be used for generating *CSSM*. For example, *x*‐ *CSSM* was generated by a single row matrix (1×2) from the *x*‐component [4 8] of the untranslated straight line points (a,b: Fig.4), if we subtracted 4 from both elements, a new (1×2) matrix would be generated [0 4], the latter could be used in generating (2×2) *x*‐ *CSSM* by keeping the zero diagonal element . The details of the method are presented in Fig.1 and Fig.2 [4].

### Calculation of allometric coefficient

The allometric coefficient is estimated for a group (sublineage) of cells in *C. elegans* in logarithmic relation to all of the cells in a developing embryo at a given development stage (time point). The data of the Cartesian coordinates were collected and the *CSSM* were generated, the *BSM* constructed and the determinants were calculated for the stages of development (time points), followed by estimation of the logarithms of the absolute values of the *BSM* determinants. A linear regression was done between the data of the cells in the lineage against the cells in developing embryo at different time points of development, while the b coefficient (slope) of the regression line was considered as the allometric coefficient ( Fig.6).

### Calculation of complexity index

The complexity was evaluated by the complexity index, which was calculated according to Chan *et al*. [152] with modification in the calculation of the branching coefficient. Three parameters were used to calculate the complexity index ( *p*_*i*_: branching coefficient, *z*_*i*_ : branching level coefficient, *C*_*i*_ : complexity coefficient). The summation of *C*_*i*_ is the complexity index. The developing embryo had an organized growth, forming a specified pattern with constant cellular arrangement. During embryo development, the time points were determined when at least one new branch (cell) was added to the morphogenesis tree at a time point. Each time point was considered as a separate level (degree) and the number of the branches was counted. The last time point (last level) provided the total number of branches. The branching points at the first time point was considered as the first level, the next time point was considered as the second level branch and so forth. After assigning the development process into different levels, the branching level coefficient *z*_*i*_ was determined, then the branching coefficient ( *p*_*i*_) was calculated after counting the number of branches at each level and divided it by the total number of branches. The complexity coefficient ( *C*_*i*_) was calculated by multiplying *p*_*i*_ with *z*_*i*_, then the complexity index was calculated by summing *C*_*i*_ values.

Fig.5 demonstrates an example for calculating the complexity index. The morphogenesis of AB blastomere was used for 2 successive generations. The zygote (P0) was divided into AB and P1, in this example, the AB blastomere was considered as the first degree (first level) of complexity containing a single branch. The division of AB into ABa and ABp was considered as the second degree (second level) of complexity containing two branches. This was followed by ABa division (third degree: third level of complexity) into ABal and ABar, while the division of ABp resulted into ABpl and ABpr, containing totally four branches. These three levels were used for estimating the complexity index. The table in Fig.5 shows the calculation of *p*_*i*_, *z*_*i*_ and *C*_*i*_ *,* while the value of the calculated complexity index was 2.125.

### Calculation of lineage growth rate

By definition, the growth rate is the increase in the number of cells as a function of time in a developing embryo [153]. The calculations proceeded as above, the logarithms of the absolute values of the *BSM* determinants were considered as the ordinate of *xy* plane plotted against time (abscissa), and the data were analyzed by nonlinear regression ( Fig.8).

### Comparison between this method with other methods

The allometric scaling used in this study was compared with other methods used for estimating the allometric coefficient to determine the allometric scaling for the whole body‐ and organ‐based allometry ( Tab.6). CSSM method was applied to the whole body‐ and organ‐based allometric scaling, which can be used in temporal studies for evaluating the biodynamic changes in size, shape, mass and surface of the whole body and organs. Tab.7 shows several methods that used allometric scaling applied to cell‐based allometry; this table compares different parameters with the parameter used in this study (cell center). Also, cell‐based allometric scaling can be used in temporal studies for evaluating the biodynamics in cell distribution and pattern, by using cell center, cell volume, position and cell surface of the differentiating and differentiated tissues as parameters in generating *CSSM*. Similar studies can be used in investigating the compartmental analysis in pharmacokinetics and pharmacodynamics of drugs and toxins. In Tab.8, we list the methods of allometric scaling used for investigating subcellular‐, molecular‐, pharmacological‐ and biochemical‐based studies. We can apply the *CSSM* for evaluating the dynamic changes of organelles distribution and density at the subcellular level, as well as at the molecular level, such as in gene regulation and expression.

### Application of the method to *C. elegans* embryo

#### Generation of *CSSM* method to *C. elegans* embryo

The Cartesian coordinates of the center of a dividing cell were extracted from the images captured from EMS (12 cells) and ABp (32 cells) lineages of a *C. elegans* embryo. The 4‐dimensional data of the embryo were explained in the materials and methods [141]. The components of the Cartesian coordinates of the cells in a given lineage were processed according to a previous publication [53], while the *CSSM*, *BSM* and the *BSM* determinants were generated, constructed and calculated, respectively.

**Table 6:** A comparison of this method *(CSSM)* with the other methods at whole body and organ levels

Refs.	Based on	Applied for	Method	Purpose
[154]	Body	Whole body	Body mass orweight	Ontogenetic, static, evolutionary allometry
[154]	Body	Whole body	Body volume	Ontogenetic, static, evolutionary allometry
[155]	Body	Whole body	Body surface	Interspecies equivalents for therapeutic dosages of drug
[75]	Organ	Liver	Mitochondrial inner membrane surface area	Cellular oxygen consumption
[156]	Organ	Heart	Electrocardiogram	Cardiovascular disease diagnosis and management of patient
[157]	Organ	Eye	Relationship between eye axial length and body weight	Eye size predictions based on the nocturnal or diurnal activity of the species
[158]	Organ	Kidney	Prediction of kidney size in reptiles	Osmoregulation
[159]	Organ	Muscle and Tendon	Allometric scaling of muscle cross‐sectional areas	Structural properties of muscle‐tendon units
[160]	Organ	Lung	Tidal volumes and breathing rates	Prediction of allometric coefficint for tidal volumes and breathing rates

Whole body‐ and organ‐based allometric scaling can be used in temporal studies for evaluating the biodynamic changes in size, shape, mass and surface of the whole body and the organs.

**Table 7:** A comparison of this method *(CSSM)* to other methods applied to the allometric scaling in cell‐based allometry

Refs.	Based on	Applied for	Method	Purpose
[10]	Cell	Female germline cyst in *Drosophila* *melanogaster*	Cell & nuclear volumes	Collective cell behavior in the female germline cyst
[161]	Cell	*In vitro*	Cell ratios	Metabolism
[162]	Cell	Embryogenesis	Cell size	Cytoskeleton
[163]	Cell	Brain cells in *Hymenoptera*	Cell number	Brain mass and nuclei number
[164]	Cell	Orbital fat	Cell number	Adipose cell number
[165]	Cell	Lung	Cell number and size	Alveolus surface area & type I, type II pneumonocyte number
[166]	Cell	Hematopoietic system	Cells Number	Number of active stem cells
Current^*^	Cell	Early embryonic development, *C. elegans*	Cell center (geometric: center of gravity)	Complexity evaluation by complexity index
[118]^**^	Cell	Early embryonic development, *C. elegans*	Cell volume	Cell cycle

This table compares different parameters with the parameters used in this study (cell center). Cell‐based allometric scaling can be used in temporal studies for evaluating the biodynamics in cell distribution and pattern using cell center, cell volume, position and cell surface of the differentiating and differentiated tissues, as parameters in generating *CSSM*. * *CSSM* means that zero‐centroaxial skew‐symmetrical matrix can be used in temporal studies for evaluating the biodynamic changes at cellular and tissue levels. The data of these parameters (in the above methods) can be entered as elements of 1×n matrix, with subsequent generation of *CSSM*. ** means power law study not allometric scaling.

**Table 8:** A comparison of our method with other methods applied to subcellular‐, molecular‐, pharmacological‐ and biochemical‐based studies

Refs.	Based on	Applied for	Method	Purpose
[167]	Subcellular	Mitochondria	Mean mitochondrial volume	O_2_ consumption relative to cell size
[162]	Subcellular	Embryogenesis	Cell size	Cytoskeleton
[168]	Molecular	Cell volume & body size	Allometric scaling of RNA abundance	Metabolic theory of ecology, growth rate hypothesis on messenger RNA and ribosome abundance
[169]	Molecular	Cell size	RNA content	Cell differentiation and proliferative capacity of rat keratinocytes
[170]	Molecular	Body size & shape ontogeny	Quantitative trait loci	Genetic architecture of ontogenetic changes in body shape and its associated allometry
[171]	Molecular	Facial shape	Quantitative trait loci	Facial diversity that is influenced by key genes in skeletal and facial development
[161]	Biochemical	*In vitro*	Cell ratios	Metabolism
[172]	Biochemical	Metabolism	Mitochondrial	Predict allometric scaling of aerobic metabolism
[75]	Biochemical	Metabolism	Mitochondrial inner membrane surface area	Cellular oxygen consumption
[173]	Biochemical	Metabolism	Basal metabolic rate (BMR)	No difference between two types ofbird
[174]	Pharmacology	Pharmacokinetics	Predict human parameters	Prediction of Irbesartan pharmacokinetic (anti‐hypertensive drug)
[175]	Pharmacology	Pharmacokinetics	Compartmental analysis	Predict the pharmacodynamic response in man
[176]	Pharmacology	Pharmacodynamics	*In vitro* preclinical study	Allometric prediction of human dose

This method *(CSSM)* can be used in evaluating the dynamic changes of organelles distribution and density at the subcellular level, as well as at the molecular level, such as in gene regulation and expression.

#### Allometric analysis

The determinants of the Cartesian coordinates components (x,y,z) at the time points were used for generating another *CSSM*, constructing the *BSM* and calculating its determinant. The logarithms of the absolute values of the *BSM* determinants (log⁡|det(BSM)|) at the developmental stages of the ABp sublineage are considered in the study (30, 55, 82, 109 and 123 minutes), the absolute values of the *BSM* determinant (log⁡|det(BSM)|) of the cells in a given time point were calculated. The logarithmic data of the cells at a given time point (abscissa) were plotted against those of the lineage (ordinate), then a linear regression was drawn and the *b* coefficients were calculated and considered as the allometric coefficients. Also, a nonlinear curve fitting (power fit model) was done using CurveExpert software 1.3 in webhop website in order to evaluate the behavior of the data.

#### Complexity index in *C. elegans*

The descent cells in the ABp and EMS sublineages were entered as branches, and the numbers of the total branches at the levels were counted. The value of *p*_*i,*_ *z*_*i,*_ *C*_*i*_ and the complexity were estimated as explained above ( Fig.5).

In the ABp sublineage, the complexity index was calculated at the branching levels, where there were 6 and 5 branching levels in ABp and EMS sublineages, respectively. The branching at each level, the total number of branches (at each level) and the total number of branches (the last level) in the ABp and EMS sublineages were determined.

#### Lineage growth rate in *C. elegans*

The data were plotted as the logarithm of the time points (abscissa) against the logarithm of the absolute values of the *BSM* determinant (log⁡|det(BSM)|) (ordinate) for the ABp and EMS sublineages. The plotted time points are 30, 55, 82, 109 and 123 minutes. The nonlinear exponential curves were fitted using CurveExpert software 1.3 in webhop website.

## COMPLIANCE WITH ETHICS GUIDELINES

The authors Ali Tarihi, Mujtaba Tarihi and Taki Tiraihi declare that they have no conflict of interest or financial conflicts to disclose.

This article does not contain any studies with human or animal materials performed by any of the authors.
